# Upfront Anti-CD38 Monoclonal Antibody-Based Quadruplet Therapy for Multiple Myeloma: A Systematic Review and Meta-Analysis of Clinical Trials

**DOI:** 10.3390/cancers17121943

**Published:** 2025-06-11

**Authors:** Ioannis Ntanasis-Stathopoulos, Charalampos Filippatos, Panagiotis Malandrakis, Vassilis Koutoulidis, Efstathios Kastritis, Evangelos Terpos, Meletios-Athanasios Dimopoulos, Maria Gavriatopoulou

**Affiliations:** 1 Department of Clinical Therapeutics, Alexandra General Hospital, School of Medicine, National and Kapodistrian University of Athens, 11528 Athens, Greece; 2 First Department of Radiology, Areteion Hospital, School of Medicine, National and Kapodistrian University of Athens, 11528 Athens, Greece; 3 Department of Medicine, Korea University, Seoul 02841, Republic of Korea

**Keywords:** quadruplet, anti-cd38, first-line, ndmm, multiple myeloma, systematic review, meta-analysis

## Abstract

**Background:** Recently, the addition of anti-CD38 monoclonal antibodies (mAbs) to standard first-line triplet regimens, including a proteasome inhibitor (PI), an immunomodulatory drug (IMiD) and dexamethasone, has led to the introduction of quadruplets in clinical practice. **Methods:** A systematic search was conducted (end-of-search: 9 November, 2024) for clinical trials investigating first-line anti-CD38 mAb-based quadruplets in combination with a PI and an IMiD. Pooled proportions and effect-estimates along with 95% confidence intervals were calculated with common-effect and random-effects models and further subgroup and meta-regression analyses were performed. **Results:** The pooled 2-, 3- and 4-year progression-free survival (PFS) rates were 89%, 77% and 86%, respectively. Furthermore, patients treated with quadruplets demonstrated a 46% reduced risk for disease progression or death (HR = 0.54, 95% CI: 0.46–0.64) compared to those on triplets. Overall survival (OS) rates were consistently high, ranging from 83% to 96% between different regimens. High rates of deep responses that deepened over time were observed, with the pooled proportion of patients achieving at least complete response being 64%. Importantly, the pooled MRD negativity rate was 62%, whereas patients treated with quadruplet first-line therapy had 2.5 times the odds to be MRD negative at any point compared with those on triplets. Moreover, the odds for sustained 12-month MRD negativity were thrice as much with quadruplets compared to triplets. Finally, while no increase in serious adverse events was observed with quadruplet regimens compared to triplets, a 46% statistically significant increased risk for grade 3–4 neutropenia and thrombocytopenia was observed, along with a 14% increased risk for grade 3–4 infections. **Conclusions:** The addition of anti-CD38 mAbs to standard triplet regimens has shown particularly favorable outcomes, supporting their integration in the upfront treatment of patients with NDMM. However, close monitoring for hematological toxicity and infections is essential.

## Simple Summary

Recent advances in treating newly diagnosed multiple myeloma (NDMM) have explored the addition of anti-CD38 antibodies to established three-drug regimens, forming quadruplets. This article reviewed and meta-analyzed 18 clinical trials involving over 4100 patients to deeply understand these four-drug combinations. The findings suggest that quadruplet regimens significantly reduce the risk of disease progression or death—by nearly half when compared to triplets—while overall demonstrating commendable survival benefits. They also led to much higher rates of deep treatment responses, as shown by minimal residual disease (MRD) negativity, including sustained MRD negativity over 12 months. However, this intensive therapeutic approach also resulted in higher rates of grade 3–4 neutropenia, thrombocytopenia, and infections. This research article seeks to support clinicians in evaluating treatment options by providing insights into the potential benefits and risks, which may help inform NDMM management.

## 1. Introduction

While multiple myeloma (MM) remains an incurable plasma cell dyscrasia, the incorporation of novel therapeutic agents that target specific pathways involved in tumor growth and survival have significantly improved patient outcomes and survival [1,2]. Anti-CD38 antibodies are a crucial component of these novel agents, demonstrating remarkable efficacy, particularly when combined with other standard anti-myeloma regimens, thus providing a valuable addition to the therapeutic arsenal for MM [3,4]. These antibodies target CD38, a cell surface protein highly expressed on malignant MM cells, leading to direct cytotoxicity, immune modulation, and enhanced anti-tumor activity [5].

Daratumumab, the first anti-CD38 monoclonal antibody to be given approval both for patients with newly diagnosed MM (NDMM) and relapsed/refractory MM (RRMM), is now a backbone of MM patient management, having shown significant efficacy when combined with other standard anti-myeloma regimens [6,7,8]. In the newly diagnosed stage of the disease, combinations of daratumumab with standard first-line regimens have led to remarkable outcomes, thus providing a variety of treatment options for NDMM patients, including both transplant-eligible and -ineligible patient populations [9,10,11].

A standard and highly effective treatment approach for NDMM is the triplet combination of a proteasome inhibitor (PI), an immunomodulatory drug (IMiD), and dexamethasone, as it has led to prolonged median progression-free survival (PFS) and overall survival (OS) outcomes [12,13,14]. The introduction of anti-CD38 monoclonal antibodies has now increased interest in quadruplet first-line regimens, as key randomized clinical trials have demonstrated deeper responses, including higher rates of minimal residual disease (MRD) negativity, as well as prolonged PFS in patients with NDMM [15,16,17]. In addition to daratumumab, isatuximab has emerged as another anti-CD38 monoclonal antibody with significant efficacy in both RRMM and NDMM settings. The recently published IMROZ study highlighted the benefits of combining isatuximab with bortezomib, lenalidomide, and dexamethasone (VRd), showing improved progression-free survival and deeper responses in NDMM patients, further supporting its role as a viable option in the first-line setting [18].

In this context, we conducted a comprehensive systematic review and meta-analysis on both randomized and non-randomized clinical trials treating patients with quadruplet induction regimens, involving an anti-CD38 monoclonal antibody, a PI, an IMiD and dexamethasone.

## 2. Materials and Methods

The present meta-analysis was performed following the Preferred Reporting Items for Systematic Reviews and Meta-Analyses (PRISMA) guidelines [19]. The study protocol was discussed and agreed upon in advance by all authors. The study protocol was registered in the Open Science Framework registry (10.17605/OSF.IO/E4RG5).

A systematic search was conducted in the PubMed database from conception until 29 November 2024 for literature on clinical trials evaluating quadruplet first-line MM therapies involving an anti-CD38 monoclonal antibody, a proteasome inhibitor, a thalidomide variant and dexamethasone.

The search algorithm implemented was as follows:

((“Multiple Myeloma”[Mesh] OR “multiple myeloma”[tiab]) AND
(“Daratumumab”[Mesh] OR “Daratumumab”[tiab] OR “DARA”[tiab]) AND
((“Bortezomib”[Mesh] OR “Bortezomib”[tiab] OR “VRD”[tiab] OR “VTD”[tiab]) OR
(“Carfilzomib”[Mesh] OR “Carfilzomib”[tiab] OR “KRD”[tiab])) AND
(“Lenalidomide”[Mesh] OR “Lenalidomide”[tiab] OR “Thalidomide”[Mesh] OR “Thalidomide”[tiab]) AND (“Dexamethasone”[Mesh] OR “Dexamethasone”[tiab]))

Eligible articles included peer-reviewed full-texts of clinical trials on quadruplet anti-myeloma therapies for NDMM, single arm or controlled with standard of care or placebo controls (no multi-arm), which reported survival outcomes (PFS and OS) either in terms of proportions or in terms of effect outcomes between arms. These were the main outcomes for this study. Secondary outcomes included key response and safety characteristics, such as rates of at least complete response (≥CR), MRD negativity rates and adverse events. Case–control, cohort and cross-sectional studies, case series and case reports, reviews, in vitro and animal studies were not included in this meta-analysis.

### 2.1. Data Abstraction and Effect Estimates

The data abstraction encompassed general information (first author’s name, publication year, database and clinical trial ID), study characteristics (phase, blinding, follow-up, geographic region, number of participants, number of males, age, risk stratification), intervention characteristics (experimental and control arm treatment), efficacy (PFS, OS, ≥CR and MRD negativity rates) and key safety outcomes (rates of serious adverse events (SAEs), neutropenia, thrombocytopenia and infections). Extracted effect estimates included hazard ratios (HRs) alongside their 95% confidence intervals (CIs) per outcome.

If one of the above was not found in the main article, the Supplementary Material was thoroughly screened. There was no shortage of required data for the purposes of the meta-analysis. Data were independently extracted, analyzed and recorded. The finalized data form was reached after team consensus.

### 2.2. Statistical Analyses

Statistical analyses included pooling of studies as well as meta-regressions. Common and Random-effects models were appropriately used to calculate the pooled effect estimates (Proportions, HRs and ORs). Between-study heterogeneity was assessed by Q-test and I^2^ estimations. When heterogeneity was not low (I^2^ > 40%), random-effect model results were deemed appropriate. Subgroup analyses were performed based on follow-up timepoint, quadruplet regimen, trial design, trial phase, NDMM setting and MM risk stratification by population. Exploratory analyses included a sub-analysis for treatment effect-estimates between arms reported for high-risk cytogenetic subgroups in the RCTs included. Post hoc meta-regression analyses were performed in order to assess whether other moderators within the study sample modified the reported effect estimates. Variables included were key study aspects that introduce heterogeneity and had 10 or more entries. Throughout the analysis *p*-values were two-sided and the significance level was 0.05. All statistical analyses were performed using R/R-Studio version 2024.04.2+764 (Posit Software, PBC).

### 2.3. Assessment of Study Quality and Risk of Bias

All records included clinical trials, either blinded or open label. Risk was assessed with the implementation of the RoB:2 and ROBINS-I V2 algorithms by Cochrane to our analysis tools [20].

Publication bias was to be evaluated in the analyses that included 10 or more study arms [21] and that did not exclusively report raw number or proportions [22,23]. For this purpose, Egger’s statistical test (statistical significance *p* < 0.1) [24] was implemented as well as the funnel plot inspection [25]. The evaluation of publication bias was performed using R/R-Studio version 2024.04.2+764 (Posit Software, PBC, Boston, MA, USA).

## 3. Results

### 3.1. Selection of Studies

**Figure 1:**
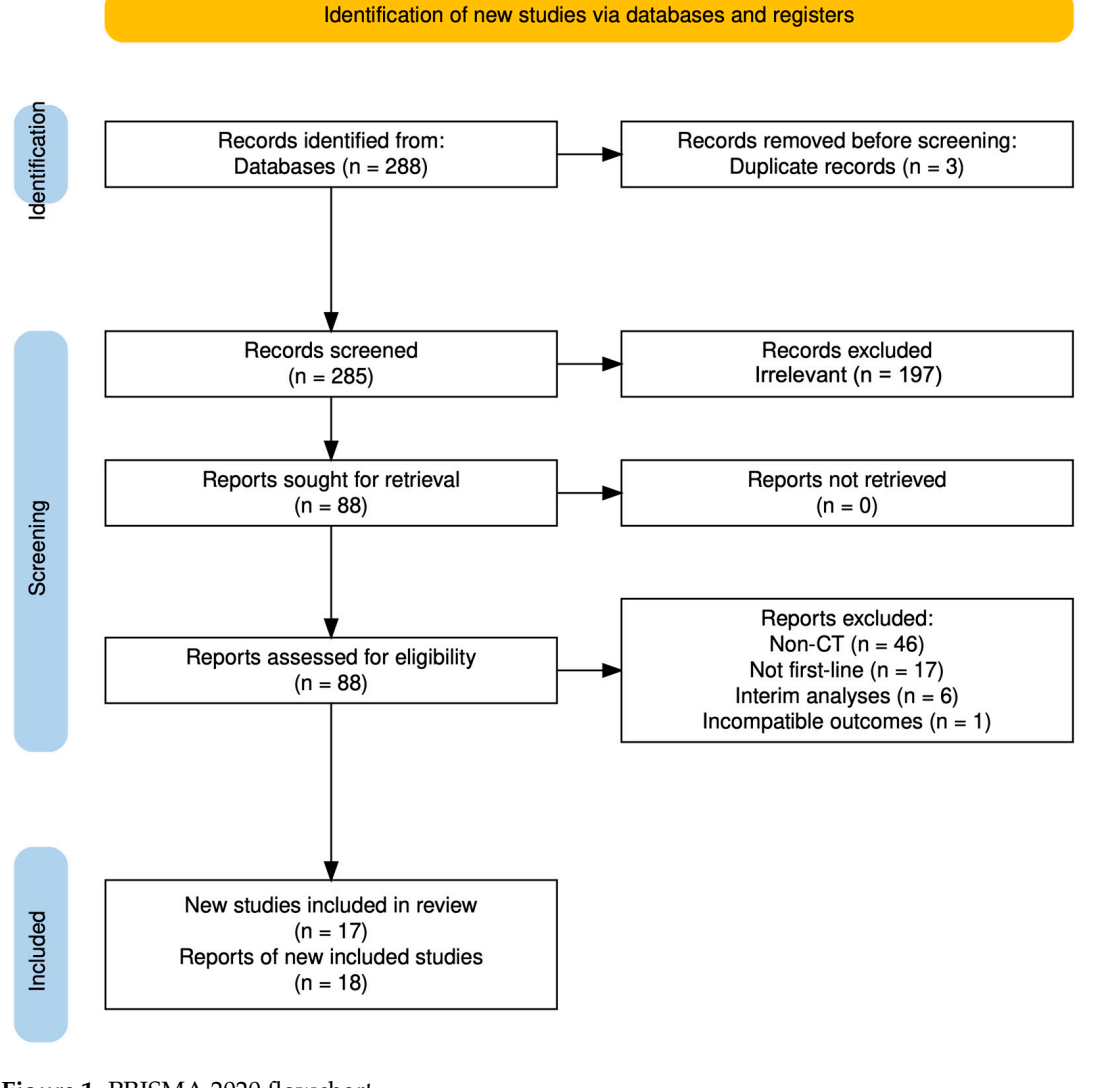
PRISMA 2020 flowchart.

A total of 288 articles were identified through the search algorithm detailed in the Materials and Methods section. Of these, 200 were excluded during the initial screening phase, leaving 88 records for full-text retrieval. Upon further evaluation, 19 articles were deemed eligible, representing 18 clinical trials investigating first-line therapies incorporating an anti-CD38 antibody, a proteasome inhibitor, a thalidomide derivative, and dexamethasone. These trials collectively involved 4100 patients [16,17,18,26,27,28,29,30,31,32,33,34,35,36,37,38,39,40]. Figure 1 illustrates the step-by-step screening process.

Out of the seventeen trials included, five were randomized controlled trials, and the remaining twelve were non-randomized. Ten trials involved transplant-eligible patients, three transplant-ineligible and four mixed populations. Table 1 portrays key baseline trial and population characteristics.

### 3.2. Progression-Free Survival

**Figure 2:**
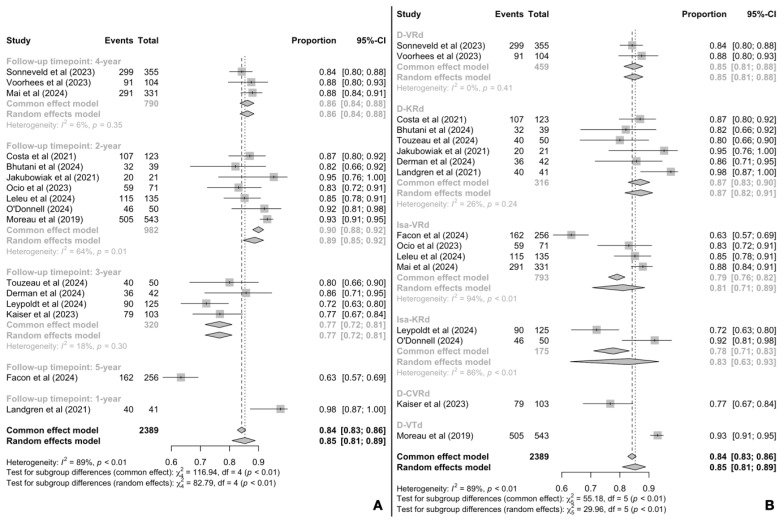
Pooled PFS rates [16,17,18,26,27,28,30,31,32,33,34,35,36,37,38,40] by (**A**) follow-up timepoints and (**B**) treatment regimen.

Fifteen records reported PFS outcomes in terms of percentages at varying timepoints (Figure 2). The overall pooled estimate was 85% (95% CI: 81–89%) survival rate without disease progression or death, while heterogeneity was particularly high (I^2^ = 89%). Subgroup analyses revealed statistically significant differences in the PFS rates reported at different timepoints (*p* < 0.01; Figure 2A) and with different regimens (*p* < 0.01; Figure 2B).

Specifically, the pooled 2-, 3- and 4-year PFS rates were 89% (95% CI: 85–92%), 77% (95% CI: 72–81%) and 86% (95% CI: 84–88%), respectively, while only one trial reported results at 1- and at 5-year timepoints. Treatment with D-KRd (daratumumab, carfilzomib, lenalidomide, dexamethasone) demonstrated a pooled PFS rate of 87% (95% CI: 83–90%), D-VRd (daratumumab, bortezomib, lenalidomide, dexamethasone) a pooled rate of 85% (95% CI: 81–88%), Isa-KRd (isatuximab, carfilzomib, lenalidomide, dexamethasone) a pooled rate of 83% (95% CI: 63–93%) and Isa-VRd (isatuximab, bortezomib, lenalidomide, dexamethasone) a pooled rate of 81% (95% CI: 71–89%).

Moreover, trials focused on high-risk MM patients demonstrated a marginally statistically significant (*p* = 0.04) lower PFS rate (79% PFS%, 95% CI: 72–85%) than trials on standard MM populations (87% PFS%, 95% CI: 82–91%) (Supplementary Table S1). Subsequent meta-regression analysis (Supplementary Table S2) revealed a statistically significant and negative association between increased age (−0.009% change for 1-year increase, *p* = 0.007) and longer follow-up times (−0.174% change for 1-month increase, *p* < 0.001).

Additionally, an exploratory multivariate meta-regression analysis aiming to adjust for the effect of induction therapy intensity and the nature of consolidation and maintenance therapy, in trials involving transplant -eligible patients, was conducted. No statistically significant associations were revealed (Supplementary Table S3).

**Figure 3:**
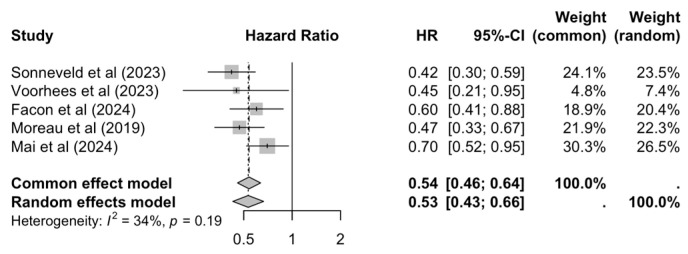
Pooled PFS HR [16,17,18,34,40] between quadruplet and triplet arms.

Furthermore, five RCTs reported PFS in terms of HRs between treatment arms, resulting in a pooled 46% reduced risk for disease progression or death for patients on a quadruplet induction regimen compared to those receiving a triplet (HR = 0.54, 95% CI: 0.46–0.64), while heterogeneity was low (I^2^ = 34%) (Figure 3).

Subgroup analyses revealed no statistically significant differences, potentially attributed to the low number of trial entries and consistent results among the included ones (Supplementary Table S4).

### 3.3. High-Risk Cytogenetic Exploratory PFS Sub-Analysis

The RCTs included also provided treatment effect-estimates between arms specifically for patients harboring high-risk cytogenetic aberrations. It was observed that patients treated with quadruplets had a 16% reduced risk for disease progression or death (HR = 0.84, 95% CI: 0.63–1.11) compared to those treated with triplets, albeit the results are non-significant (Supplementary Figure S1).

### 3.4. Overall Survival

**Figure 4:**
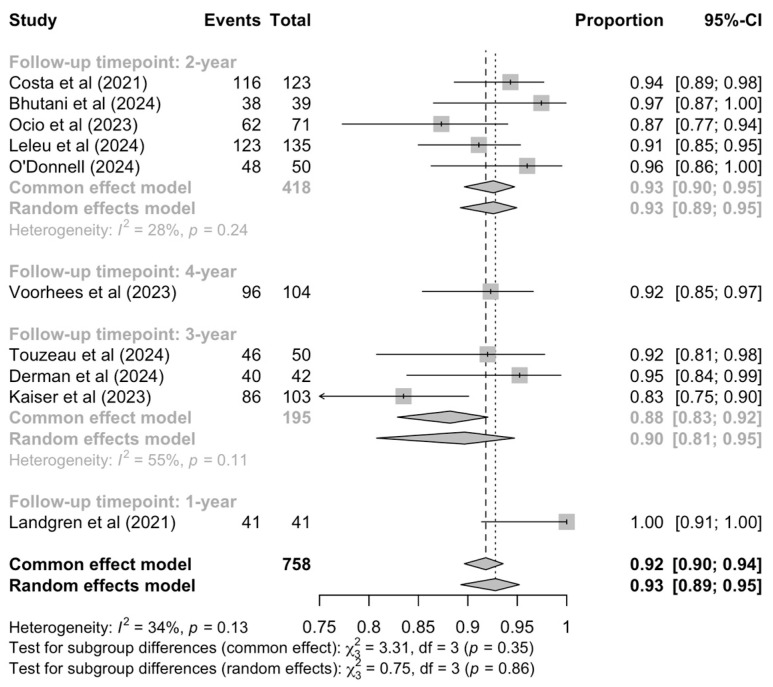
Pooled OS rates [16,26,27,28,31,33,35,36,37,38] by follow-up timepoint.

Ten studies reported OS outcomes as percentages at varying timepoints (Figure 4). The pooled analysis revealed an overall 92% (95% CI: 90–94%) survival rate with low heterogeneity (I^2^ = 34%). Subgroup analyses by prespecified key characteristics revealed statistically significant differences in the OS rates with different quadruplet regimens (*p* < 0.01) and marginally non-statistically significant differences between transplant-eligible and -ineligible patients (*p* = 0.06) (Supplementary Table S5).

Per pooled estimates, patients treated with D-KRd demonstrated a 95% OS rate (95% CI: 92–97%) and those treated with Isa-VRd a 90% (95% CI: 85–93%). Only one trial for each of D-VRd, D-CVRd and Isa-KRd regimens reported OS rates, ranging from 83% to 96%. Meta-regression analysis identified no potential covariates (Supplementary Table S6).

### 3.5. Complete Response Rates

Seventeen records reported CR or better rates (≥CR), as part of the best response to first-line therapy. The pooled overall ≥CR rate at first-line therapy with quadruplets was 64% (95% CI: 52–75%), while heterogeneity was particularly high (I^2^ = 96%) (Supplementary Figure S2, Supplementary Table S7).

Pooled estimates revealed that responses deepened over time (*p* < 0.001), as the 2-year, 3-year and 4-year ≥CR rates were 58% (95% CI: 45–71%), 62% (95% CI: 45–76%) and 75% (95% CI: 49–90%), respectively. At the ½-year, 1-year and 5-year follow-up timepoints, there was only one trial and ≥CR rates were 16%, 95% and 75%, respectively.

Regarding the variability among responses in different quadruplet regimens (*p* < 0.001), the pooled ≥CR rates in trials on D-VRd, D-KRd, Isa-VRd and Isa-KRd were 66% (95% CI: 23–92%), 75% (95% CI: 58–87%), 55% (95% CI: 41–68%) and 66% (95% CI: 59–73%), respectively. For the D-CVRd and D-VTd regimens, the ≥CR rates were 49% and 39%, respectively, in one trial each. No significant associations were identified in meta-regression analyses (Supplementary Table S8).

In six trials with control arms, the calculation of ORs for ≥CR was possible and per pooled effect estimate, patients treated with quadruplet induction regimens had twice the odds to demonstrate a complete response or better (OR = 2.08, 95% CI: 1.61–2.70) compared to those treated with standard triplets without anti-CD38 monoclonal antibodies (Supplementary Figure S3, Supplementary Table S9).

### 3.6. Minimal Residual Disease Negativity

Records from fifteen trials reported the number of patients who were MRD-negative at any point during first-line therapy, with the pooled percentage being 62% (95% CI: 53–70%) with high heterogeneity (I^2^ = 85%) (Supplementary Figure S4). Subgroup analyses revealed statistically significant differences between different trial phases, NDMM settings and MM risk (Supplementary Table S10).

Treatment with D-KRd resulted in a pooled MRD negativity rate of 70% (95% CI: 47–86%), Isa-KRd in a pooled rate of 69% (95% CI: 49–83%), Isa-VRd in a rate of 52% (95% CI: 47–57%) and D-VRd in a rate of 51% (95% CI: 31–71%). Subsequent meta-regression analysis did not identify any significant moderators (Supplementary Table S11).

**Figure 5:**
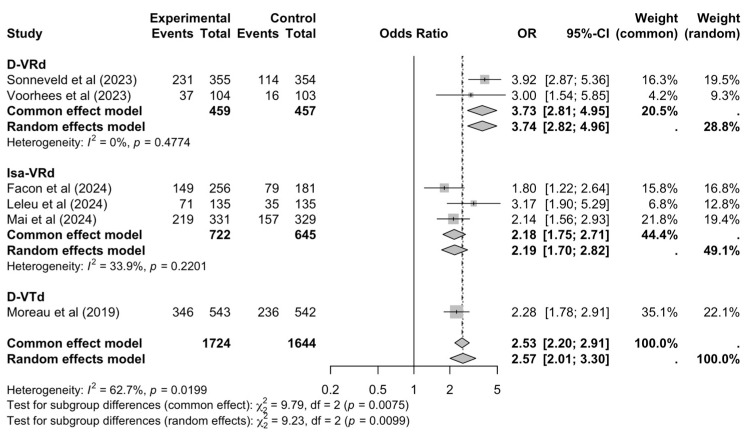
Pooled MRD negativity OR [16,17,18,34,36,40], by treatment regimen.

Furthermore, in six trials with triplet control arms, calculations of ORs for head-to-head comparisons were possible. Per overall pooled estimate, patients treated with quadruplet first-line therapies had as many as 2.5 times the odds to be MRD-negative at any point (OR = 2.57, 95% CI: 2.01–3.30) compared to those treated with standard triplets, while heterogeneity was high (I^2^ = 63%) (Figure 5, Supplementary Table S12).

Moreover, four of the RCTs included reported outcomes for sustained 12-month MRD negativity. Patients who were treated with quadruplet regimens exhibited as many as 3 times the odds to sustain MRD negativity (OR = 3.04, 95% CI: 2.18–4.26) compared to those treated with triplets (Supplementary Figure S5).

### 3.7. Safety

**Figure 6:**
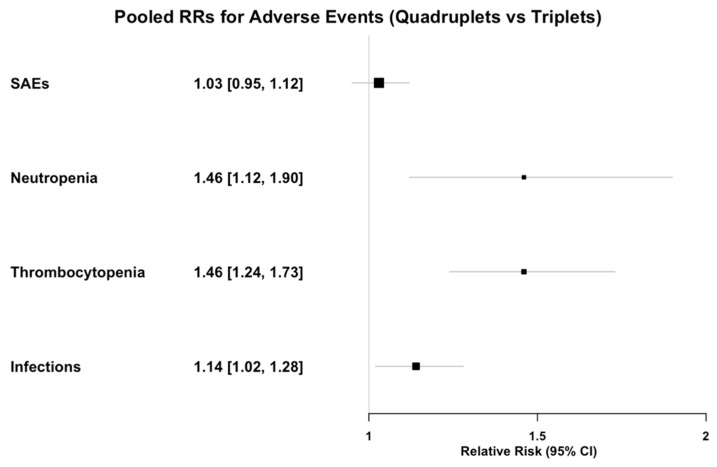
Pooled RRs for adverse events between quadruplets and triplets.

The assessment of safety outcomes encompassed SAEs, neutropenia, thrombocytopenia and infections expressed as relative risks (RRs) between arms, in order to compare quadruplet and triplet regimens in this regard. A total of five controlled studies were included in this sub analysis (Supplementary Figures S6–S9). Per pooled analysis, quadruplet first-line therapy was not linked with an increased risk for SAEs (RR = 1.03, 95% CI = 0.95–1.12); however, it was linked with a 46% increased risk for grade 3–4 neutropenia (RR = 1.46, 95% CI: 1.12–1.90), a 46% increased risk for grade 3–4 thrombocytopenia (RR = 1.46, 95% CI: 1.24–1.73) and a 14% increased risk for grade 3–4 infections (RR = 1.14, 95% CI: 1.02–1.28) (Figure 6).

### 3.8. Risk of Bias Assessment

The risk of bias across included studies varied by study design. All RCTs were assessed using the RoB:2 tool and demonstrated an overall low risk of bias, with some concerns noted specifically in the domain of missing outcome data related to MRD analyses (Supplementary Table S13).

In contrast, non-randomized studies, assessed using the ROBINS-I tool, generally exhibited moderate risk of bias with some serious risk exceptions, primarily due to confounding of interventions, and in some cases missing outcome data (Supplementary Table S14). Single-arm trials were particularly susceptible to these limitations, underscoring the inherent challenges in drawing causal inferences from non-randomized evidence.

## 4. Discussion

Until recently, triplet regimens had been the standard of care for patients with NDMM, demonstrating consistent improvements in patient outcomes in both clinical trials and real-world settings [41,42,43]. Anti-CD38 mAb-based therapies have shown significant benefits in the treatment of relapsed/refractory multiple myeloma (RRMM), with robust improvements in PFS and OS across numerous studies [44,45,46,47]. As these anti-CD38 therapies are now being incorporated into frontline quadruplet regimens, it becomes increasingly important to evaluate their efficacy and safety profiles comprehensively.

This meta-analysis, encompassing data from 18 clinical trials and 4100 NDMM patients, showed significant benefits from the addition of anti-CD38 mAbs to potent first-line triplets including a PI and an IMiD. The overall pooled PFS and OS rates were 85% and 93%, ranging from 63% to 98% and 83% to 97% at different timepoints, respectively. Pooled rates of ≥CR and MRD negativity during first-line therapy were also remarkable, at 64% (range 23–95%) and 62% (range 36–94%). Among controlled trials, patients treated with quadruplets (a) exhibited a pooled 46% reduced risk for disease progression or death, (b) double the odds for a ≥CR, (c) had as many as 2.5 times the odds to be MRD-negative at any point and (d) exhibited as many as 3 times the odds to sustain MRD negativity for 12 months, when compared to those treated with triplets. Results were also consistent in trials enrolling high-risk multiple myeloma patients (HRMM).

While the enhanced efficacy of quadruplet therapy is evident, its adoption in routine practice requires careful patient selection and proactive monitoring for treatment-related toxicities, particularly infections and hematologic adverse events. Pooled results from controlled trials showed no statistically significant differences in overall grade 3–4 SAEs when comparing quadruplets to triplets, but there were increased risks for grade 3–4 neutropenia, thrombocytopenia and infections. These findings underscore the importance of implementing infection prophylaxis measures as NDMM patients bear an increased risk of infections due to a combination of factors [48]. In the COVID-19 era, vaccination and oral antivirals were essential for the prophylaxis of MM patients, while tixagevimab/cilgavimab and convalescent plasma were proven to have low value against newer variants [49,50,51]. Vaccination, in general, is especially crucial for MM patients due to their increased vulnerability, which stems both from the immune system compromise caused by the disease itself and the immunosuppressive effects of their treatments [52].

The results of our analysis are in line with the 59% reduced risk for disease progression or death for patients treated with quadruplets compared to those with triplets, shown in a small-scale meta-analysis on transplant-eligible patients with NDMM [53]. They are also consistent with the results from a meta-analysis focusing only on RCTs that showed a 45% reduced risk for progression or death, a 21% increased probability of ≥CR, a 39% increased probability of MRD negativity at the 10^−5^ threshold and similar risks for adverse events, when comparing quadruplets to triplets [54]. It is noteworthy that the latter work included two RCTs currently not published as peer-reviewed full texts, the IsKia and GEM2017FIT trials [55,56].

At IsKia, in NDMM transplant-eligible patients treated with Isa-KRd vs. KRd over a median follow-up of 20 months, marginally statistically significant increased odds for MRD negativity were observed in the quadruplet arm (OR = 1.67, *p* = 0.0049), whereas ≥CR rates (74% vs. 72%) and PFS rates (both 95% at 1-year) were similar [56]. GEM2017FIT enrolled elderly, non-transplant-eligible NDMM patients who were treated with D-KRd, KRd or VMP-Rd (control). The MRD negativity rate for the D-KRd arm was 79% vs. 69% for KRd, while ≥CR rates (61% vs. 59%) and PFS rates (both 87% at 18-months) were comparable [56].

A randomized trial involving NDMM patients not undergoing upfront ASCT that has not been included in any meta-analysis and not published as full text yet is the CEPHEUS study. In the latest abstract presented, over a median follow-up of 58.7 months, D-VRd overall MRD negativity odds were twice as high; both the 10^−5^ and 10^−6^ thresholds and odds for sustained 12-month MRD negativity were 2.63 higher, with D-VRd vs. VRd [57]. These observations are similar to our pooled-effect estimates.

Results for transplant-eligible NDMM patients were further validated and studied in a large comparative real-world analysis of D-VRd versus VRd involving 1000 patients [58]. Those treated with the quadruplet regimen, compared to the triplet, showed a statistically significant 67% and 47% reduced risk in PFS and OS events, respectively, albeit no differences in ≥CR rates were noted. Another study in Korea highlighted the advantage of achieving deep responses after quadruplet induction therapy compared to triplet, as patients on D-VTd compared to those on VRd exhibited higher MRD negativity rates (94.4% vs. 66.7%), at least very good partial response (VGPR) rates (93.0% vs. 67.6%) and ≥CR rates (90.5% vs. 68.5%) [59].

At this point, it shall be noted that the heterogeneity and variability in the differences observed among the ≥CR rates are potentially attributable to daratumumab interfering with the serum immunofixation assay and the variability of methods implemented among the included trials to compensate for this effect when assessing the response outcomes [60,61].

The parallel improvement in survival outcomes and MRD negativity rates observed in our meta-analysis and the aforementioned studies emphasizes the robust efficacy of anti-CD38 monoclonal antibody-based quadruplet regimens for NDMM. This finding is particularly significant given recent evidence supporting the use of MRD negativity as a surrogate endpoint for treatment efficacy in multiple myeloma [62,63]. It is worth noting that the high heterogeneity observed in the MRD results across the included studies is potentially linked to the different techniques used, sensitivity thresholds and timepoints of assessment [64].

The present meta-analysis, compared to previously conducted ones, is the only one to our knowledge that includes both randomized and non-randomized clinical trials, enabling a broader spectrum of outcomes, including both transplant-eligible and -ineligible NDMM patients and combined populations. Moreover, the inclusion of trials exclusively involving quadruplet regimens comprising all three drug classes—anti-CD38 mAbs, PIs, and IMiDs—alongside dexamethasone, ensured universal consistency across this analysis. Another notable strength of this analysis is the comprehensive subgroup analyses performed, enabling detailed comparisons between different groups to account for variability in study characteristics and their populations. On the other hand, limitations of this study include the high heterogeneity observed during the assessment of outcomes such as survival rates or MRD negativity, the considerable risk of bias from missing information regarding MRD assessment, the limited number of controlled trials and the limited number of trials at some follow-up timepoints. Specifically, for MRD, such gaps introduce potential bias, limiting the generalizability and interpretability of findings and, thus, necessitating cautious consideration of the synthesized and pooled results. Moreover, the strength of this meta-analysis in the inclusion of non-randomized also poses limitations, as single-arm studies carried generally a moderate to serious risk of bias. These inherent design constraints may reduce the certainty of effect estimates and limit the strength of causal inferences compared to randomized controlled trials. Finally, heterogeneity in definitions of high-risk cytogenetics between trials may affect subgroup interpretations.

## 5. Conclusions

Our meta-analysis, involving data from 18 clinical trials and 4100 NDMM patients, provides robust evidence supporting the efficacy of anti-CD38 mAb-based quadruplet regimens as first-line therapy for NDMM. By incorporating anti-CD38 mAbs, PIs, IMiDs and steroids, these quadruplets yield remarkable outcomes and deliver significant improvements in survival and in the depth of responses compared to standard triplets. The findings underscore the potential of quadruplet regimens to redefine the treatment paradigm for NDMM in both transplant-eligible and transplant-ineligible patients. Future research should focus on refining therapeutic strategies, optimizing toxicity management, and further validating the observed benefits in populations with high-risk cytogenetics and poor prognosis.

## Figures and Tables

**Table 1:** Trial and baseline population characteristics.

Author (Year)	RCT ^§^	Phase	ASCT ^§^	HRMM ^§^	Regimen	Age (yrs ^§^)	Patients	FUP (mos ^§^)
BENEFIT Leleu et al. (2024) [36]	Yes	3	TIE **^§^**	No	Isa-VRd **^§^**	61	270	23.5
CASSIOPEIA Moreau et al. (2019) [40]	Yes	3	TE **^§^**	No	D-VTd **^§^**	59	1085	18.8
GMGG-CONCEPT Leypoldt et al. (2024) [39]	No	2	Any	Yes	Isa-KRd **^§^**	62	125	41.7
GMGG-HD7 Mai et al. (2024) [34]	No	3	TE	Yes	Isa-VRd	59	660	48
GRIFFIN Voorhees et al. (2023) [16]	Yes	2	TE	No	D-VRd **^§^**	59	207	49.6
IFM 2018-04 Touzeau et al. (2024) [28]	No	2	TE	Yes	D-KRd **^§^**	57	50	33
IMROZ Facon et al. (2024) [18]	Yes	3	TIE	No	Isa-VRd	72	437	59.7
MANHATTAN Landgren et al. (2021) [37]	No	2	Any	No	D-KRd	59	41	11
MASTER Costa et al. (2021) [26]	No	2	TE	Yes	D-KRd	60	123	25.1
NCT01998971 Jakubowiak et al. (2021) [30]	No	1	TE	No	D-KRd	59.5	21	23.3
NCT02513186 Ocio et al. (2023) [35]	No	1b	TIE	No	Isa-VRd	71	71	24
NCT03500445 Derman et al. (2024) [31]	No	2	Any	No	D-KRd	58	42	27
NCT04113018 Bhutani et al. (2024) [27]	No	2	Any	No	D-KRd	-	39	30.1
OPTIMUM Kaiser et al. (2023) [33]	No	2	TE	Yes	D-CVRd **^§^**	60	103	30
PERSEUS Sonneveld et al. (2023) [17]	Yes	3	TE	No	D-VRd	61	709	47.5
SKylaRk O’ Donnell et al. (2024) [38]	No	2	TE	No	Isa-KRd	59	50	26

^§^ RCT = randomized controlled trial, ASCT = autologous stem cell transplantation, HRMM = high-risk MM, NDMM = newly diagnosed multiple myeloma, TE = transplant-eligible, TIE = transplant-ineligible, D = daratumumab, Isa = isatuximab, V = bortezomib, K = carfilzomib, R = lenalidomide, T = thalidomide, d = dexamethasone, FUP = follow-up, yrs = years, mos = months.

## Data Availability

Data available upon reasonable request.

## Supplementary Materials

The following supporting information can be downloaded at: https://www.mdpi.com/article/10.3390/cancers17121943/s1, Figure S1: Exploratory PFS sub-analysis per high-risk cytogenetics; Figure S2: Meta-analysis of ≥CR %, by quadruplet regimen; Figure S3: Meta-analysis of ≥CR ORs, by follow-up timepoint; Figure S4: Meta analysis of MRD-%, by quadruplet regimen; Figure S5: Pooled OR for sustained 12-month MRD negativity; Figure S6: Pooled RR for grade 3–4 SAEs; Figure S7: Pooled RR for grade 3–4 neutropenia; Figure S8: Pooled RR for grade 3–4 thrombocytopenia; Figure S9: Pooled RR for grade 3–4 infections; Table S1: Subgroup analysis of PFS% meta-analysis; Table S2: Meta-regression analysis for PFS% meta-analysis; Table S3: Exploratory multivariate meta-regression analysis for the effect of induction, consolidation and maintenance therapy on transplant-eligible populations; Table S4: Subgroup analysis of PFS HRs meta-analysis; Table S5: Subgroup analysis of OS proportions meta-analysis; Table S6: Meta-regression analysis for OS% meta-analysis; Table S7: Subgroup analysis of ≥CR % meta-analysis; Table S8: Meta-regression analysis for ≥CR % meta-analysis; Table S9: Subgroup analysis of ≥CR ORs meta-analysis; Table S10: Subgroup analysis of MRD negativity rates meta-analysis; Table S11: Meta-regression analysis for MRD-% meta-analysis; Table S12: Subgroup analysis of MRD negativity ORs meta-analysis; Table S13: Risk of bias assessment, RCTs (RoB:2); Table S14: Risk of bias assessment, non-randomized trials (adapted ROBINS-I)
