# Supplementary material for: Upfront Anti-CD38 Monoclonal Antibody-Based Quadruplet Therapy for Multiple Myeloma: A Systematic Review and Meta-Analysis of Clinical Trials
Source: Cancers (Basel). 2025 Jun 11;17(12):1943. doi: 10.3390/cancers17121943 (PMC12190497; doi:10.3390/cancers17121943)
Supplement: Supplementary file 1 [file cancers-17-01943-s001.zip › cancers-3653591-supplementary.pdf]

Supplementary Table S1. Subgroup analysis of PFS% meta-analysis

**Subgroup**	**#**	**Pooled estimate**	**95% CI**	**I^2**	**p-value**
All (overall)	15	85%	80% - 89%	89%	-
**Timepoint**					**< 0.01**
1-year	1	98%	87% - 100%	-	
2-year	7	90%	85% - 92%	64%	
3-year	4	77%	72% - 81%	18%	
4-year	2	85%	81% - 88%	0%	
5-year	1	63%	57% - 69%	-	
**Regimen**					**< 0.01**
D-VRd	2	85%	81% - 88%	0%	
D-KRd	6	87%	83% - 90%	26%	
D-VTd	1	93%	91% - 95%	-	
D-CVRd	1	77%	67% - 84%	-	
Isa-VRd	3	78%	65% - 87%	92%	
Isa-KRd	2	83%	63% - 93%	86%	
Setting					0.22
NDMM TE	8	87%	83% - 91%	79%	
NDMM TIE	3	78%	65% - 87%	92%	
NDMM (any)	4	86%	72% - 93%	70%	
Trial					0.92
Randomized	5	85%	75% - 91%	96%	
Non-randomized	10	85%	79% - 89%	59%	
Phase					0.63
1	1	95%	76% - 100%	-	
1b	1	83%	72% - 81%	-	
2	4	85%	79% - 89%	64%	
3	9	84%	71% - 91%	97%	
Population					0.05
Any	11	87%	81% - 91%	91%	
High-risk	4	79%	72% - 85%	64%	

Supplementary Table S2. Meta-regression analysis for PFS% meta-analysis

**Covariate**	**Increment**	**Change in proportion**	**p-value**
Age (median, yrs)	1-year increase	-0.009%	0.007
Follow-up (median, months)	1-month increase	-0.174%	<0.001

Supplementary Table S3. Exploratory multivariate meta-regression analysis for the effect of induction, consolidation and maintenance therapy on transplant-eligible populations

**Covariate**	**Change in proportion**	**p-value**	**Adjusted I^2**
# of induction cycles (≤ 4)	0.013%	0.114	-
Consolidation			
None (reference)	-	-	-
Anti-CD38-based	-0.008%	0.623	-

PI-IMiD-based	<0.001%	0.957	-
Maintenance<br>None<br>Anti-CD38-based<br>IMiD-based	 -<br>-0.055%<br>-0.084%	 -<br>0.357<br>0.289	 -<br>-<br>-
Analysis timepoint (1-year increase)	-0.009%	0.054	-
-	-	-	0.00%

Supplementary Table S4. Subgroup analysis of PFS HRs meta-analysis

Subgroup	#	Pooled estimate	95% CI	I^2	p-value
All (overall)	4	0.48	0.39 – 0.59	0%	-
Timepoint<br>2-year<br>4-year<br>5-year	 1<br>2<br>1	 0.47<br>0.42<br>0.60	 0.33 – 0.67<br>0.31 – 0.58<br>0.41 – 0.88	 -<br>0%<br>-	0.38
Regimen<br>D-VRd<br>D-VTd<br>Isa-VRd	 2<br>1<br>1	 0.42<br>0.47<br>0.60	 0.31 – 0.58<br>0.33 – 0.67<br>0.41 – 0.88	 0%<br>-<br>-	0.38
Setting<br>NDMM TE<br>NDMM TIE	 3<br>1	 0.44<br>78%	 0.35 – 0.56<br>65% - 87%	 0%<br>-	0.19
Phase<br>2<br>3	 1<br>3	 0.45<br>0.48	 0.21 – 0.95<br>0.39 – 0.59	 -<br>0%	0.86

Supplementary Figure S1. Exploratory PFS sub-analysis per high-risk cytogenetics

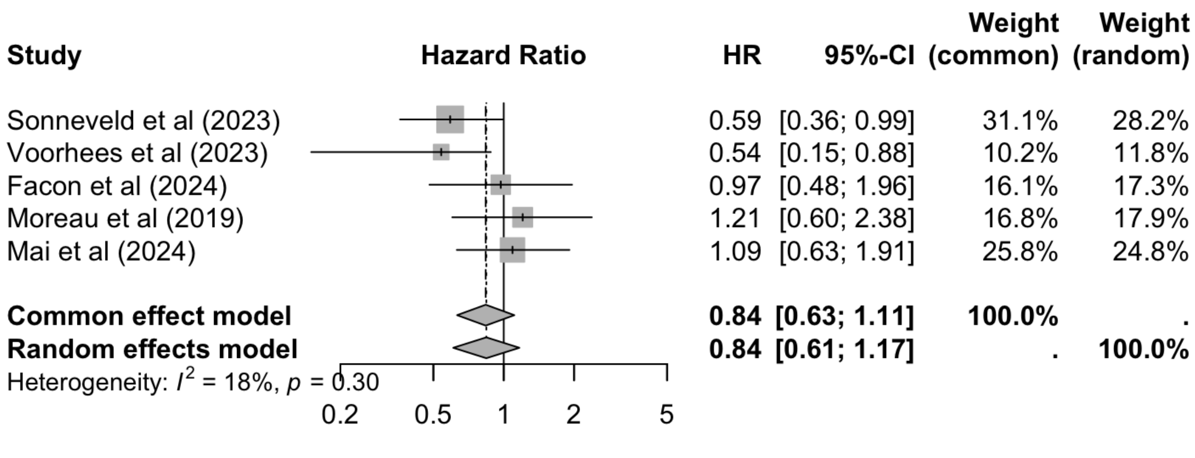

Supplementary Table S5. Subgroup analysis of OS proportions meta-analysis

Subgroup	#	Pooled estimate	95% CI	I^2	p-value
All (overall)	10	92%	90% - 94%	34%	-
**Timepoint**<br>1-year	 1	 100%	 91% - 100%	 -	0.86

2-year<br>3-year<br>4-year	5<br>3<br>1	93%<br>90%<br>92%	90% - 95%<br>81% - 95%<br>85% - 97%	28%<br>55%<br>-	
**Regimen**					**< 0.01**
D-VRd	1	92%	85% - 97%	-	
D-KRd	5	95%	92% - 97%	0%	
D-CVRd	1	83%	75% - 90%	-	
Isa-VRd	2	90%	85% - 93%	0%	
Isa-KRd	1	96%	86% - 100%	-	
Setting					0.06
NDMM TE	4	92%	87% - 95%	59%	
NDMM TIE	2	90%	85% - 93%	0%	
NDMM (any)	3	98%	93% - 99%	0%	
Trial					0.95
Randomized	8	94%	89% - 96%	46%	
Non-randomized	2	93%	89% - 95%	0%	
Phase					0.20
1b	1	87%	77% - 94%	-	
2	8	94%	90% - 97%	45%	
3	1	91%	85% - 95%	-	
Population					0.35
Any	7	93%	90% - 95%	0%	
High-risk	3	90%	83% - 95%	75%	

Supplementary Table S6. Meta-regression analysis for OS% meta-analysis

**Covariate**	**Increment**	**Change in proportion**	**p-value**
Age (median, yrs)	1-year increase	-0.014%	0.190
Follow-up (median, months)	1-month increase	-0.081%	0.293

Supplementary Figure S2. Meta-analysis of ≥CR %, by quadruplet regimen

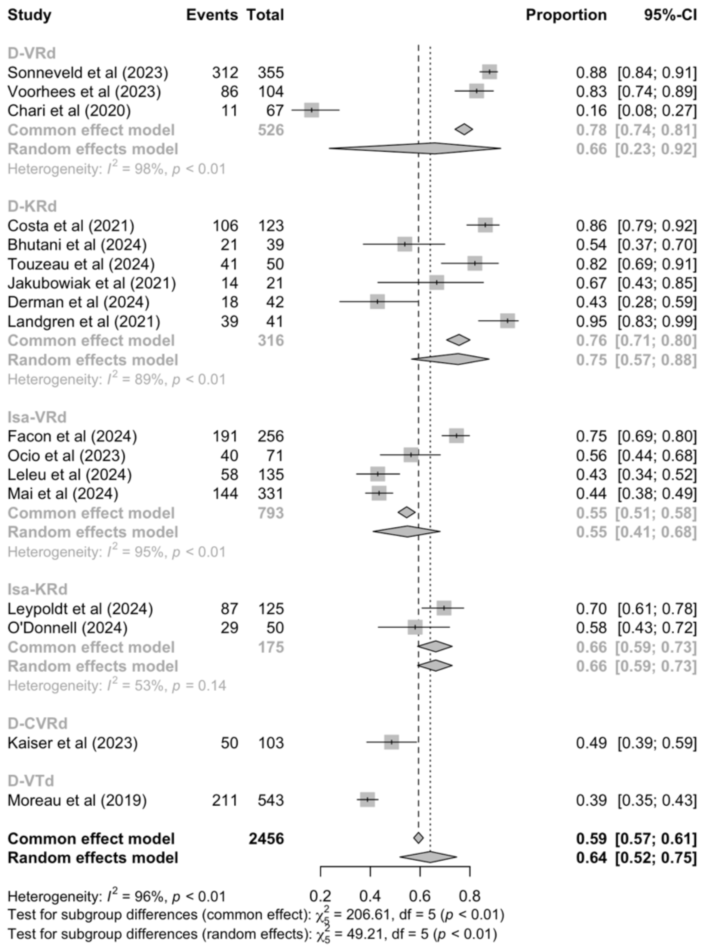

Supplementary Table S7. Subgroup analysis of ≥CR % meta-analysis

**Subgroup**	**#**	**Pooled estimate**	**95% CI**	**I^2**	**p-value**
All (overall)	17	63%	50% - 74%	34%	-
**Timepoint**					**<0.01**
½-year	2	23%	19% - 27%	47%	
1-year	1	95%	83% - 99%	-	
2-year	7	58%	45% - 71%	92%	
3-year	4	62%	45% - 76%	88%	
4-year	2	87%	83% - 90%	46%	
5-year	1	75%	69% - 80%	-	
**Regimen**					**< 0.01**
D-VRd	3	66%	23% - 92%	98%	
D-KRd	6	75%	58% - 87%	89%	
D-CVRd	1	49%	39% - 59%	-	
D-VTd	1	39%	35% - 43%	-	
Isa-VRd	4	49%	30% - 69%	98%	
Isa-KRd	2	66%	59% - 73%	53%	
Setting					0.92
NDMM TE	10	61%	43% - 77%	98%	
NDMM TIE	3	59%	43% - 73%	95%	

NDMM (any)	4	70%	43% - 88%	86%	
Trial					0.53
Randomized	5	68%	48% - 84%	98%	
Non-randomized	12	60%	44% - 75%	95%	
Phase					0.69
1	1	67%	43% - 85%	-	
1b	1	56%	44% - 68%	-	
2	10	67%	49% - 81%	93%	
3	5	56%	32% - 77%	99%	
Population					0.90
Any	12	93%	90% - 95%	96%	
High-risk	5	64%	49% - 82%	98%	

Supplementary Table S8. Meta-regression analysis for ≥CR % meta-analysis

**Covariate**	**Increment**	**Change in proportion**	**p-value**
Age (median, yrs)	1-year increase	0.557%	0.778
Follow-up (median, months)	1-month increase	0.914%	0.009

Supplementary Figure S3. Meta-analysis of ≥CR ORs, by follow-up timepoint

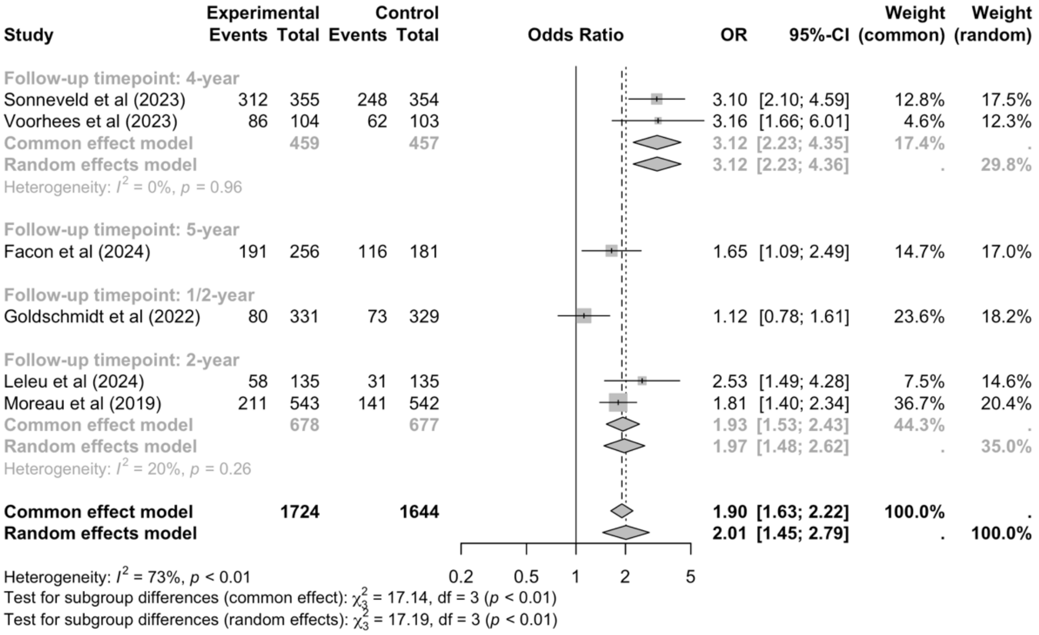

Supplementary Table S9. Subgroup analysis of ≥CR ORs meta-analysis

**Subgroup**	**#**	**Pooled estimate**	**95% CI**	**I^2**	**p-value**
All (overall)	6	2.01	1.45 – 2.79	73%	-
**Timepoint**					**<0.01**
½-year	1	1.12	0.78 – 1.61	-	
2-year	2	1.93	1.53 – 2.43	20%	
4-year	2	3.12	2.23 – 4.35	0%	
5-year	1	1.65	1.09 – 2.49	-	

**Regimen**					**< 0.01**
D-VRd	2	3.12	2.23 – 4.36	0%	
D-VTd	1	1.81	1.40 – 2.34	-	
Isa-VRd	3	1.62	1.03 – 2.54	69%	
Setting					0.92
NDMM TE	4	2.04	1.26 – 3.31	82%	
NDMM TIE	2	1.90	1.41 – 2.69	36%	
Trial					**<0.01**
Randomized	5	2.25	1.72 – 2.96	53%	
Non-randomized	1	1.12	0.78 – 1.61	-	
Phase					0.17
2	1	3.16	1.66 – 6.01	-	
3	5	1.89	1.34 – 2.67	75%	
Population					**<0.01**
Any	5	2.25	1.72 – 2.96	53%	
High-risk	1	1.12	0.78 – 1.61	-	

Supplementary Figure S4. Meta analysis of MRD-%, by quadruplet regimen

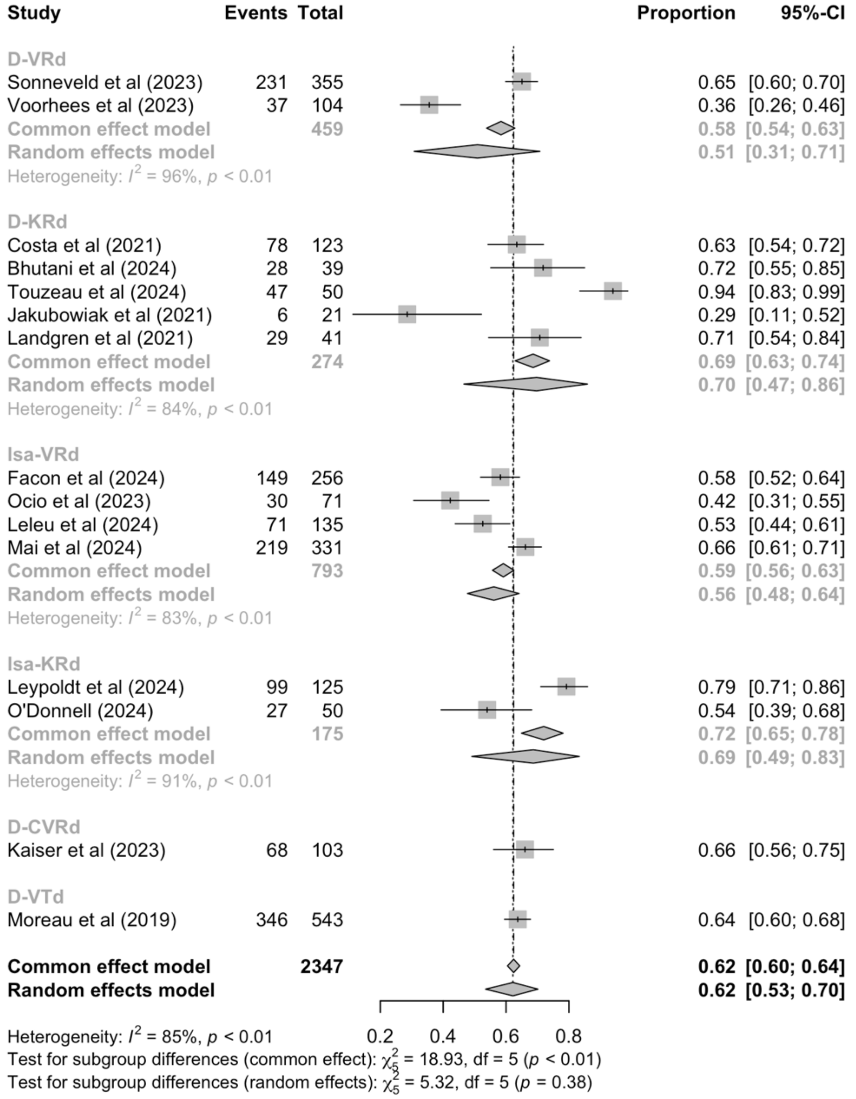

Supplementary Table S10. Subgroup analysis of MRD negativity rates meta-analysis

Subgroup	#	Pooled estimate	95% CI	I^2	p-value
All (overall)	15	61%	52% - 69%	87%	-
**Timepoint**					**<0.01**
½-year	1	50%	45% - 56%	-	
1-year	1	71%	54% - 84%	-	
2-year	7	56%	47% - 64%	77%	
3-year	3	81%	64% - 92%	85%	
4-year	2	51%	31% - 71%	96%	
5-year	1	58%	52% - 64%	-	
**Regimen**					**< 0.01**
D-VRd	2	51%	31% - 71%	96%	
D-KRd	5	70%	47% - 86%	84%	
D-CVRd	1	66%	56% - 75%	-	
D-VTd	1	64%	60% - 68%	-	
Isa-VRd	4	52%	47% - 57%	57%	
Isa-KRd	2	69%	49% - 83%	91%	
Setting					**< 0.01**
NDMM TE	9	60%	46% - 72%	88%	
NDMM TIE	3	53%	46% - 60%	65%	
NDMM (any)	3	76%	70% - 81%	0%	
Trial					0.28
Randomized	5	56%	47% - 65%	88%	
Non-randomized	10	64%	51% - 75%	87%	
Phase					**< 0.01**
1	1	29%	11% - 52%	-	
1b	1	42%	31% - 55%	-	
2	8	69%	55% - 80%	89%	
3	5	58%	53% - 64%	83%	
Population					0.06
Any	10	55%	47% - 63%	84%	
High-risk	5	61%	52% - 69%	92%	

Supplementary Table S11. Meta-regression analysis for MRD-% meta-analysis

Covariate	Increment	Change in proportion	p-value
Age (median, yrs)	1-year increase	0.201%	0.347
Follow-up (median, months)	1-month increase	0.055%	0.854

Supplementary Table S12. Subgroup analysis of MRD negativity ORs meta-analysis

Subgroup	#	Pooled estimate	95% CI	I^2	p-value
All (overall)	6	2.50	1.90 – 3.29	69%	-
**Timepoint**					**<0.01**
½-year	1	1.82	1.33 – 2.49	-	
2-year	2	2.42	1.94 – 3.02	23%	

4-year	2	3.73	2.81 – 4.95	0%	
5-year	1	1.80	1.22 – 2.64	-	
**Regimen**					**< 0.01**
D-VRd	2	3.74	2.81 – 4.95	0%	
D-VTd	1	2.28	1.78 – 2.91	-	
Isa-VRd	3	2.01	1.62 – 2.50	46%	
Setting					0.74
NDMM TE	4	2.50	1.90 – 3.29	77%	
NDMM TIE	2	2.33	1.34 – 4.04	67%	
Trial					0.08
Randomized	5	2.69	2.00 – 3.63	67%	
Non-randomized	1	1.82	1.33 – 2.49	-	
Phase					0.59
2	1	3.00	1.54 – 5.85	-	
3	5	2.45	1.81 – 3.32	75%	
Population					0.08
Any	5	2.62	2.00 – 3.63	67%	
High-risk	1	1.82	2.33 – 2.49	-	

Supplementary Figure S5. Pooled OR for sustained 12-month MRD negativity

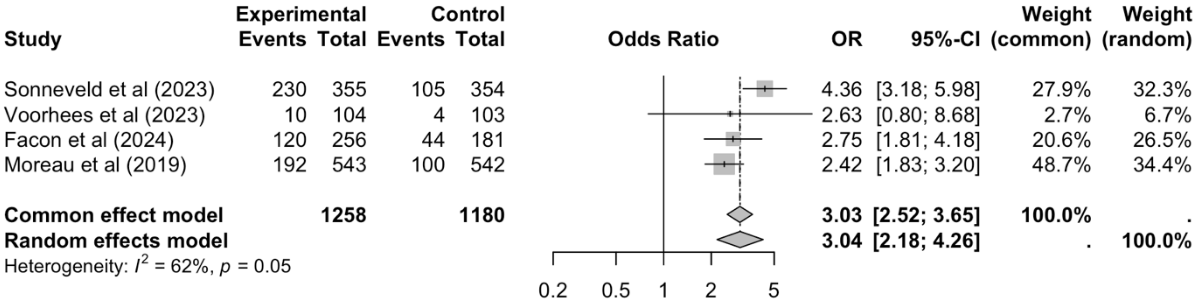

Supplementary Figure S6. Pooled RR for grade 3-4 SAEs

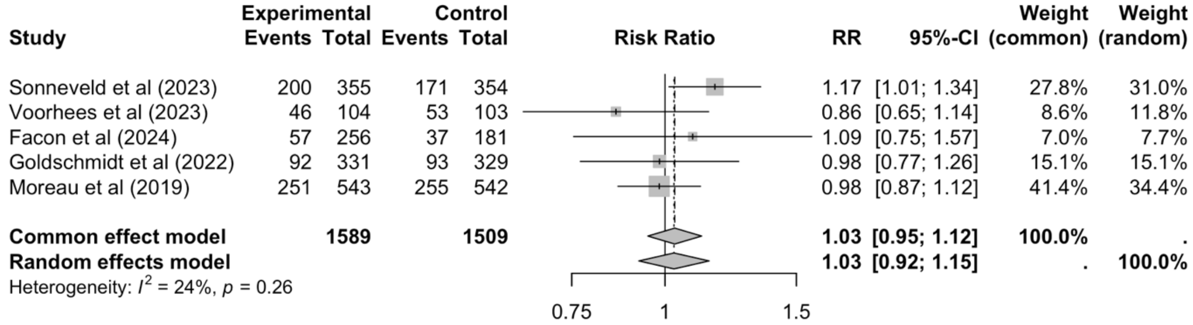

Supplementary Figure S7. Pooled RR for grade 3-4 neutropenia

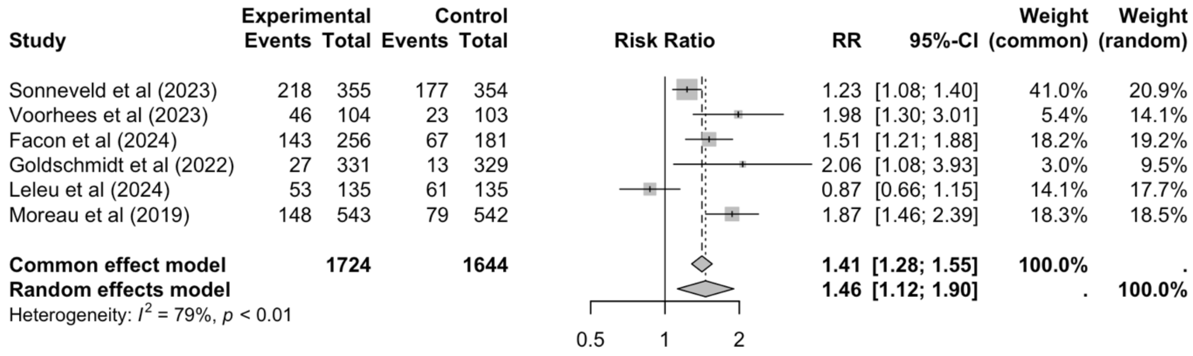

Supplementary Figure S8. Pooled RR for grade 3-4 thrombocytopenia

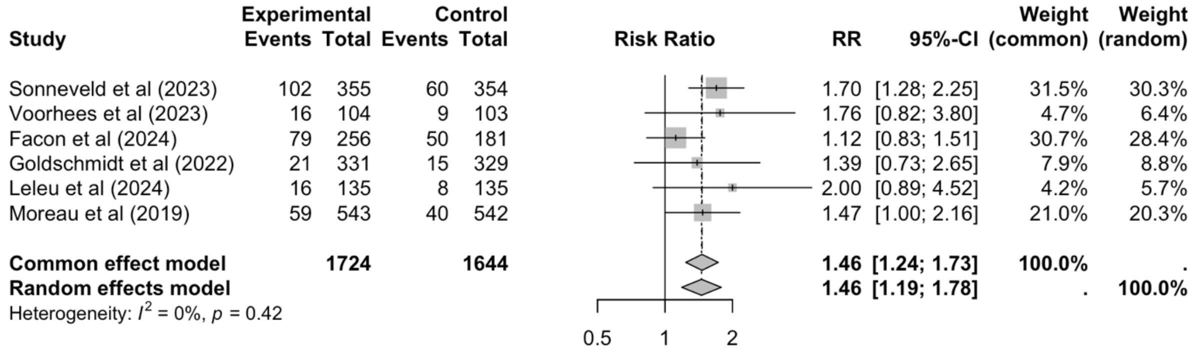

Supplementary Figure S9. Pooled RR for grade 3-4 infections

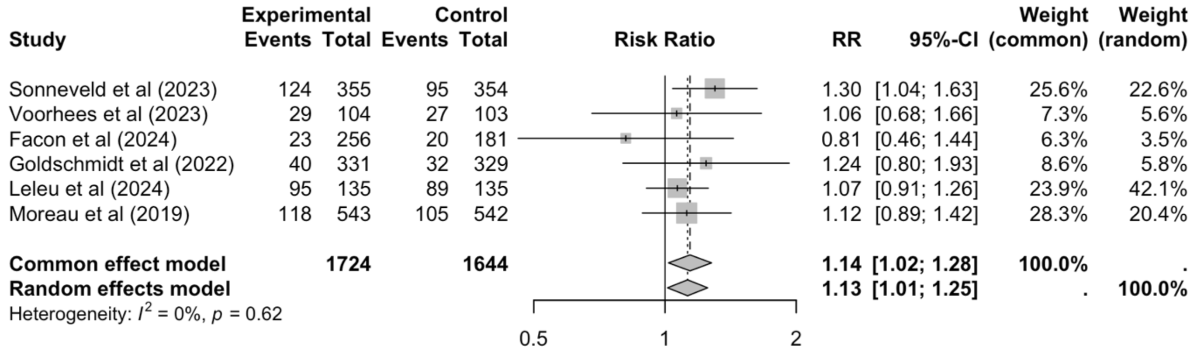

Supplementary Table S13. Risk of bias assessment, RCTs (RoB:2)

	**Randomization process**	**Deviations from the intended interventions**	**Missing outcome data**	**Measurement of the outcome**	**Selection of the reported result**	**Overall**
Mai et al (2022)	Low	Low	Low/Some concerns[a]	Low	Low	Low
Moreau et al (2019)	Low	Low	Low/Some concerns[a]	Low	Low	Low
Facon et al (2024)	Low	Low	Low/Some concerns[a]	Low	Low	Low

Leleu et al (2024)	Low	Low	Low/Some concerns[a]	Low	Low	Low
Sonneveld et al (2023)	Low	Low	Low/Some concerns[a]	Low	Low	Low
Voorhees et al (2023)	Low	Low	Low/Some concerns[a]	Low	Low	Low

[a] Some concerns in Missing Data are for the MRD analysis

Supplementary Table S14. Risk of bias assessment, non-randomized trials (adapted ROBINS-I)

**Author (year)**	**Bias due to confounding**	**Bias in classification of interventions**	**Bias in selection of participants**	**Bias due to deviations from intended interventions**	**Bias due to missing data**	**Bias in measurement of the outcome**	**Bias in selection of the reported result**	**Overall risk of bias**
Bhutani et al (2024)	Moderate risk	Serious risk	Moderate risk	Low risk	Low risk	Low risk	Low risk	Serious risk
Touzeau et al (2024)	Low risk	Low risk	Low risk	Low risk	Moderate risk	Low risk	Low risk	Moderate risk
Derman et al (2024)	Moderate risk	N/A	Low risk	Moderate risk	Low risk	Low risk	Low risk	Moderate risk
Leypoldt et al (2024)	Low risk	Low risk	Low risk	Low risk	Moderate risk	Low risk	Low risk	Moderate risk
O’ Donnell et al (2024)	Moderate risk	N/A	Low risk	Moderate risk	Low risk	Low risk	Low risk	Moderate risk
Kaiser et al (2023)	Moderate risk	N/A	Moderate risk	Low risk	Moderate risk	Low risk	Low risk	Moderate risk
Ocio et al (2023)	Serious risk	N/A	Low risk	Moderate risk	Low risk	Moderate risk	Low risk	Serious risk
Costa et al (2021)	Moderate risk	N/A	Low risk	Low risk	Low risk	Low risk	Low risk	Moderate risk
Jakubowiak et al (2021)	Serious risk	N/A	Low risk	Low risk	Moderate risk	Low risk	Low risk	Serious risk
Landgren et al (2021)	Moderate risk	N/A	Low risk	Low risk	Low risk	Low risk	Low risk	Moderate risk
